# The regulation of positive and negative emotions through instructed causal attributions in lifetime depression – A functional magnetic resonance imaging study

**DOI:** 10.1016/j.nicl.2018.10.025

**Published:** 2018-10-25

**Authors:** Leonie A.K. Loeffler, Sina Radke, Ute Habel, Rastko Ciric, Theodore D. Satterthwaite, Frank Schneider, Birgit Derntl

**Affiliations:** a Department of Psychiatry, Psychotherapy and Psychosomatics, Medical Faculty, RWTH Aachen, Pauwelsstrasse 30, 52074, Germany; b JARA-Institute Brain Structure Function Relationship, Research Center Jülich, RWTH Aachen, Pauwelsstrasse 30, 52074, Germany; c Institute of Neuroscience and Medicine 10, Research Center Jülich, 52425 Jülich, Germany; d Neuropsychiatry Division, Department of Psychiatry, Perelman School of Medicine, University of Pennsylvania, 3400 Spruce Street, Philadelphia, PA, USA; e Department of Psychiatry and Psychotherapy, Medical School, University of Tübingen, Calwerstrasse 14, 72076 Tübingen, Germany; f Werner Reichardt Center for Integrative Neuroscience, University of Tübingen, Otfried-Müller-Strasse 25, 72076 Tübingen, Germany; g LEAD Graduate School and Research Network, University of Tübingen, Gartenstrasse 29, 72074 Tübingen, Germany

**Keywords:** Emotion regulation, Depression, Attribution, Positive emotions, Precuneus, fMRI, Resting-state connectivity

## Abstract

Adequate emotional control is essential for mental health. Deficiencies in emotion regulation are evident in many psychiatric disorders, including depression. Patients with depression show, for instance, disrupted neural emotion regulation in cognitive regulation regions such as lateral and medial prefrontal cortices. Since depressed individuals tend to attribute positive events to external circumstances and negative events to themselves, modifying this non-self-serving attributional style may represent a promising regulation strategy. Spontaneous causal attributions are generally processed in medial brain structures, particularly the precuneus. However, so far no study has investigated neural correlates of instructed causal attributions (e.g. instructing a person to intentionally relate positive events to the self) and their potential to regulate emotions. The current study therefore aimed to examine how instructed causal attributions of positive and negative events affect the emotional experience of depressed individuals as well as its neural bases. For this purpose pictures of sad and happy faces were presented to 26 patients with a lifetime major depression (MDD) and 26 healthy controls (HC) during fMRI. Participants should respond naturally (“view”) or imagine that the person on the picture was sad/happy because of them (“internal attribution”) or because something else happened (“external attribution”). Trait attributional style and depressive symptoms were assessed with questionnaires to examine potential influential factors on emotion regulation ability.

Results revealed that patients compared to controls show a non-self-serving trait attributional style (i.e. more external attributions of positive events and more internal attributions of negative events). Intriguingly, when instructed to apply specific causal attributions during the emotion regulation task, patients and controls were similarly able to regulate positive and negative emotions. Regulating emotions through instructed attributions (internal/external attribution>view) generally engaged the precuneus, which was correlated with patients' trait attributional style (i.e. more precuneus activation during external>view was linked to a general tendency to relate positive events to external sources). Up-regulating happiness through internal (compared to external) attributions recruited the parahippocampal gyrus only in controls. The down-regulation of sadness (external>internal attribution), in contrast, engaged the superior frontal gyrus only in patients. Superior frontal gyrus activation thereby correlated with depression severity, which implies a greater need of cognitive resources for a successful regulation in more severely depressed. Patients and controls did not differ in activation in brain regions related to cognitive emotion regulation or attribution. However, results point to a disturbed processing of positive emotions in depression. Interestingly, increased precuneus resting-state connectivity with emotion regulation brain regions (inferior parietal lobule, middle frontal gyrus) was linked to healthier attributions (i.e. external attributions of negative events) in patients and controls. Adequate neural communication between these regions therefore seem to facilitate an adaptive trait attributional style. Findings of this study emphasize that despite patients' dysfunctional trait attributional style, explicitly applying causal attributions effectively regulates emotions. Future research should examine the efficacy of instructed attributions in reducing negative affect and anhedonia in depressed patients, for instance by means of attribution trainings during psychotherapy.

## Highlights

• Depressed patients show a non-self-serving trait attributional style.
• When instructed to use specific causal attributions, patients can regulate emotions.
• Emotion regulation through instructed attributions engages the precuneus.
• Precuneus activation and resting-state connectivity is linked to trait attributions.

## 1 Introduction

Dysfunctional emotion regulation is one of the core symptoms of numerous mental disorders, including major depressive disorder (Aldao et al., 2010). Insights into the emotion regulation process in depression can sharpen our understanding of its pathophysiology and improve treatment and prevention. An effective strategy for influencing emotional experiences targets individuals' interpretations of situations (Webb et al., 2012), a key component of cognitive behavioral therapy (Beck et al., 1979). Particularly in social situations, how one explains the causes of behaviors and events, might strongly influence emotional reactions, future expectations, and motivations (Abramson et al., 1978; Weiner et al., 1982). As patients with major depressive disorder (MDD), compared to healthy controls (HC), typically attribute negative events more frequently to themselves and positive events more frequently to external circumstances (Kestemont et al., 2015; Sweeney et al., 1986), these causal attributions are a promising target for influencing emotions. Recent meta-analyses signify a cognitive emotion regulation network that is engaged when individuals intentionally change how they think about a situation (“reappraisal”). This network includes dorsolateral and ventrolateral prefrontal cortices as well as temporal and parietal regions (Kohn et al., 2014; Morawetz et al., 2017). The ventrolateral prefrontal cortex (VLPFC) is thereby suggested to initiate the appraisal of a stimulus while the dorsolateral prefrontal cortex (DLPFC) processes the regulation itself. Parietal regions (e.g. angular gyrus, precuneus) together with temporal and limbic regions participate in the generation of the regulated emotion (Kohn et al., 2014). Patients with MDD show alterations in the recruitment of several of these brain regions (Beauregard et al., 2006; Johnstone et al., 2007; Kanske et al., 2012; Rive et al., 2013). In a review, Rive et al., (2013) revealed, for example, compromised lateral and medial prefrontal functioning in MDD during cognitive emotion regulation. Depression is, however, not only associated with altered brain activation, but also characterized by disturbed communication between cognitive control regions (Kaiser et al., 2016; Stange et al., 2017). A recent meta-analysis on seed-based resting-state connectivity, for instance, links major depressive disorder to an imbalanced connectivity among networks subserving emotion regulation: the authors identified hypoconnectivity between the affective network and the default network (e.g. the medial prefrontal cortex) as well as altered connectivity between ventral attention network seeds and the precuneus. Moreover, the authors found reduced connectivity within the frontoparietal network (i.e. between dorsolateral prefrontal cortex and posterior parietal cortex), which is involved in the regulation of emotion and attention. Similarly, Stange et al. (2017) revealed attenuated connectivity within the cognitive control network (e.g. dorsolateral prefrontal cortex, inferior parietal lobule, dorsal anterior cingulate cortex) in individuals with remitted MDD and linked such disrupted connectivity to a dysfunctional attributional style. Alterations in connectivity may therefore underlie cognitive phenotypes of depression and relate to patients' deficits in emotion regulation (Kaiser et al., 2016; Stange et al., 2017).

Most research on cognitive emotion regulation in depression applied reappraisal strategies that involve distancing from an emotional situation (e.g. looking at an image from a camera perspective) or imagining that an emotional scene gets better/worse. Changing causal attributions (e.g. relating positive events to the self) represents a further reappraisal strategy, which involves more self-related processing. Research showed that in both depressed patients and controls, making causal attributions relies on a fronto-temporo-parietal network (Hao et al., 2015; Kestemont et al., 2015; Seidel et al., 2012). In particular, the precuneus, a region linked to self-referential processing and understanding the causes of social behavior, seems to be critical for attribution (Cabanis et al., 2013; Grimm et al., 2009; Kestemont et al., 2015; Seidel et al., 2010). Seidel et al. (2010), for instance, asked participants to imagine social events (e.g. “a friend ignored you”) and indicate whether they would relate the cause of these events to themselves or to external sources, thereby revealing that precuneus activation differentiated external and internal attributions. Attribution research, however, merely assessed neural correlates of freely made attributions but not their potential influence on emotions. Interestingly, both traditional reappraisal strategies and freely made attributions recruit the precuneus, which suggests that emotion regulation through instructed causal attributions may also rely on this particular brain region.

**Fig. 1:**
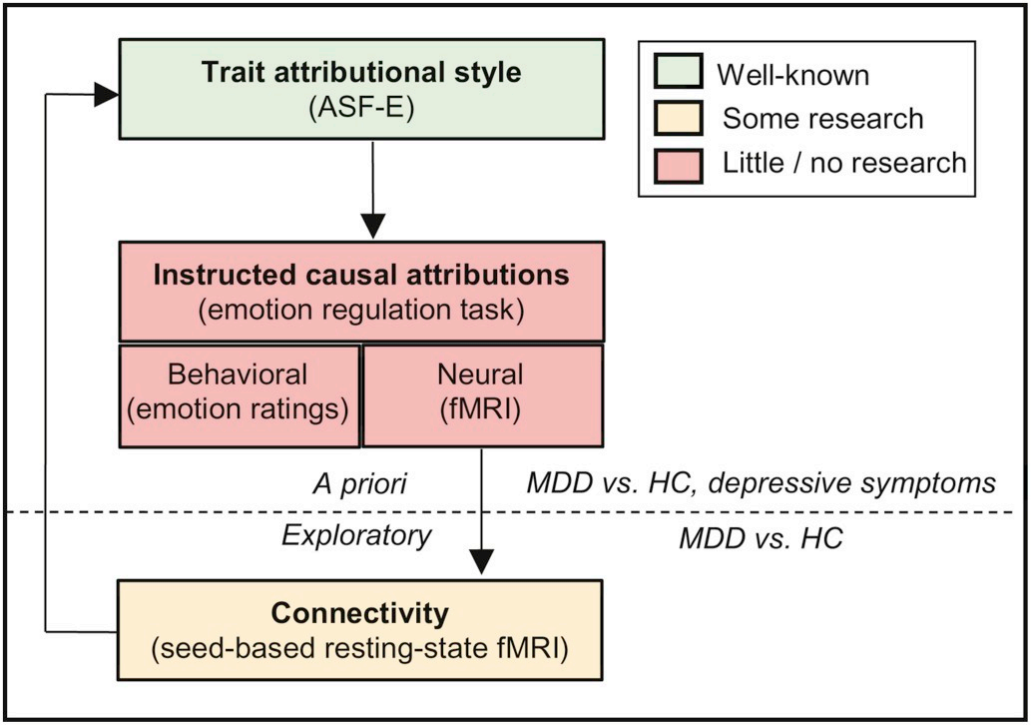
Conceptual framework. The current study examines trait attributional style, using the attributional style questionnaire (ASF-E), in patients with major depressive disorder (MDD) and healthy controls (HC). Furthermore, the influence of instructed causal attributions on subjective emotion ratings and on brain activation is investigated with an emotion regulation task during fMRI. Group differences in trait attributional style and instructed causal attributions as well as correlations with depression severity are calculated. Moreover, the relationship between trait attributional style and instructed causal attributions is examined. In addition to these a priori research aims, the study explores seed-based resting-state fMRI connectivity of brain regions identified during the emotion regulation task. Group-differences in resting-state fMRI connectivity and its association with trait attributional style are calculated.

To fill this gap of research, we examined the behavioral and neural correlates of cognitive emotion regulation by means of instructed causal attributions using fMRI and compared individuals with MDD with a healthy control group. First of all, we predicted a non-self-serving attributional style (i.e. attributing negative events to the self and/or positive events to external sources) in patients compared to controls. Such (non-self-serving) attributional style was further expected to relate to depressive symptoms and to emotion regulation (i.e. subjective emotion ratings and brain activation during emotion regulation). Second, we expected that instructed causal attributions regulate subjective emotions. Third, during emotion regulation (i.e. internal/external attribution > no regulation; internal attribution positive > external attribution positive; external attribution negative > internal attribution negative), we expected activation in brain structures related to self-referential processing such as the precuneus (Cabanis et al., 2013; Hao et al., 2015; Seidel et al., 2012) as well as in the cognitive emotion regulation network, which is generally activated during reappraisal (Kohn et al., 2014; Carmen Morawetz et al., 2017). Fourth, MDD compared to HC were expected to show impaired emotion regulation, reflected in differences in subjective emotion ratings during regulation (i.e. a reduced effect of causal attributions on subjective emotion ratings) as well as in compromised brain activation in the aforementioned brain regions (i.e. medial and lateral prefrontal cortex, precuneus). In addition to these a priori hypotheses, we aimed to explore resting-state connectivity of brain regions which we identified during the emotion regulation task and predicted attenuated connectivity in MDD compared to HC. Furthermore, attenuated connectivity in patients was expected to be linked to a dysfunctional attributional style (i.e. more internal attributions of negative events and/or more external attributions of positive events; see Fig. 1 for the conceptual framework of the study).

## 2 Methods

### 2.1 Participants

The final sample comprised 26 patients with a current (*n* = 10) or remitted (*n* = 16) major depressive disorder (MDD) according to the DSM-IV, as well as 26 healthy controls (HC) who were individually matched for age (+/− 2 years) and sex (see Table 1 for sociodemographic characteristics of the participants). Four participants (HC) were excluded from the initial sample due to technical problems during data acquisition (*n* = 1), massive head movement during scanning (*n* = 1), and non-compliance with task instructions (*n* = 2). All participants were Caucasian.

**Table 1:** Sociodemographic and clinical characteristics of study participants.

	HC (*n* = 26)	Range	MDD (*n* = 26)	Range	*p*-values
Sex (M:F)	12:14		12:14		
Age (in years)	35.31 (11.23)	22–54	35.27 (11.03)	22–55	0.956
Education (in years)	13.62 (3.03)	9–18	13.92 (3.31)	9–21	0.779
TMT-A (in seconds)	22.22 (5.81)	11–34	18.77 (4.67)	13–30	0.322
TMT-B (in seconds)	39.50 (10.06)	25–66	39.12 (15.09)	18–76	0.914
WST	31.96 (3.54)	21–36	33.42 (3.48)	24–42	0.184
MASQ	43.92 (9.93)	26–63	67.96 (17.80)	34–101	<0.001***
BDI-II	2.42 (3.20)	0–11	17.62 (10.33)	2–37	<0.001***
HRSD	–	–	10.6 (7.70)		–
STAI-State	32.04 (5.42)	24–49	40.23 (7.38)	26–60	<0.001***
STAI-Trait	33.42 (7.15)	23–58	52.35 (9.93)	33–65	<0.001***
ERQ-Reappraisal	29.04 (5.43)	12–37	23.69 (7.31)	8–35	0.008**
ERI-Reappraisal	9.12 (2.61)	5–16	6.27 (3.05)	1–14	0.001***
ASF-E Positive Internal	41.19 (4.62)	31–47	34.54 (7.07)	21–51	<0.001***
ASF-E Negative Internal	35.81 (7.04)	21–53	40.04 (8.10)	27–56	0.050*
		
Clinical state (acute/remitted)			10/16		
Time since last episode (in months)			7.15 (10.34)	0–31	
Age at onset (in years)			26.35 (9.67)	13–47	
Time since first episode (in years)			9.59 (8.54)	0.5–35	
Number of episodes			3.32 (3.30)	1–13	
Duration of episodes (in months)			6.20 (4.37)	0.75–18	
Patients with recurrent MDE			18		
SSRI only			5		
Tricyclic antidepressant only			4		
SSRI + NaSSA			3		
SSNRI only			3		
Melatonergic antidepressant only			2		
Atypical antidepressant only			1		
SSNRI + anticonvulsant			1		
SSNRI + melatonergic antidepressant			1		
SSNRI + tricyclic antidepressant			1		
NaSSA + tricyclic antidepressant			1		

Data indicated as n or mean with SD in parentheses. Abbreviations: ASF-E = Attributional Style Questionnaire for Adults, German version; BDI-II = Beck Depression Inventory-II; MDD = Patients with major depressive disorder; ERI = Emotion Regulation Inventory; ERQ = Emotion Regulation Questionnaire; HC = Healthy Controls; HRSD = Hamilton Rating Scale of Depression; MASQ = Mood and Anxiety Symptom Scale; MDE = Major Depressive Episode; NaSSA = Noradrenergic and Specific Serotonergic Antidepressant; SSNRI = Selective Serotonin Noradrenalin Reuptake Inhibitor; SSRI = Selective Serotonin Reuptake Inhibitor; STAI = State Trait Anxiety Inventory; TMT-A/B = Trail Making Test-A/B; WST = “Wortschatztest” (verbal intelligence). Group differences are indicated by *p*-values. The following significance levels are applied: *p ≤ .05, **p ≤ .01, ***p ≤ .001.

Patients were recruited from the Department of Psychiatry, Psychotherapy and Psychosomatics of the RWTH Aachen University. Exclusion criteria for patients were previous/current psychotic and (hypo-)manic symptoms, current substance dependency, treatment for other psychiatric disorders than depression, and personality disorders. HC who met criteria for any current/lifetime psychiatric disorder, or had first-degree relatives with any psychotic or bipolar disorder, were excluded. Exclusion criteria for both groups were age < 18 or > 55 years, neurological diseases, left-handedness, and contraindications for MRI.

All participants gave written informed consent and received financial compensation (30€). This study was approved by the local ethics committee of the Medical Faculty of the RWTH Aachen University and was conducted according to the Declaration of Helsinki.

### 2.2 Clinical assessment

In order to specify current clinical state participants completed measures of depression and anxiety (Beck Depression Inventory II, BDI-II (Hautzinger et al., 1995); Hamilton Rating Scale of Depression 17-items, HDRS (Williams, 1988); Mood and Anxiety Symptom Scale, MASQ (Watson and Clark, 1991); State Trait Anxiety Inventory, STAI (Laux et al., 1981)). Furthermore, all participants completed measures assessing trait emotion regulation (Emotion Regulation Questionnaire, ERQ (Abler and Kessler, 2009); Emotion Regulation Inventory, ERI (König, 2011)) and underwent neuropsychological testing tapping executive functioning (TMT-A/-B (Army Individual Test Battery, 1944)) and verbal intelligence (Wortschatztest, WST (Schmidt and Metzler, 1992)) in order to compare groups on a more general level of functioning (see Table 1 for clinical characteristics of study participants as well as group comparisons in clinical parameters).

### 2.3 Trait attributional style (ASF-E)

We assessed trait attributional style using the improved German version of the attributional style questionnaire (ASF-E (Poppe et al., 2005)). The ASF-E examines three dimensions of an attributional style (i.e. external vs. internal, unstable vs. stable, and specific vs. global attributions) for both negative and positive events. The internality dimension shows whether a person attributes the cause of an event to the self or to external circumstances/other persons and was the focus of the current study. By means of the ASF-E we could disentangle trait attributional style (i.e. how participants explain causes of events in daily life) from instructed causal attributions (i.e. during the emotion regulation task).

### 2.4 Instructed causal attributions (emotion regulation task)

**Fig. 2:**
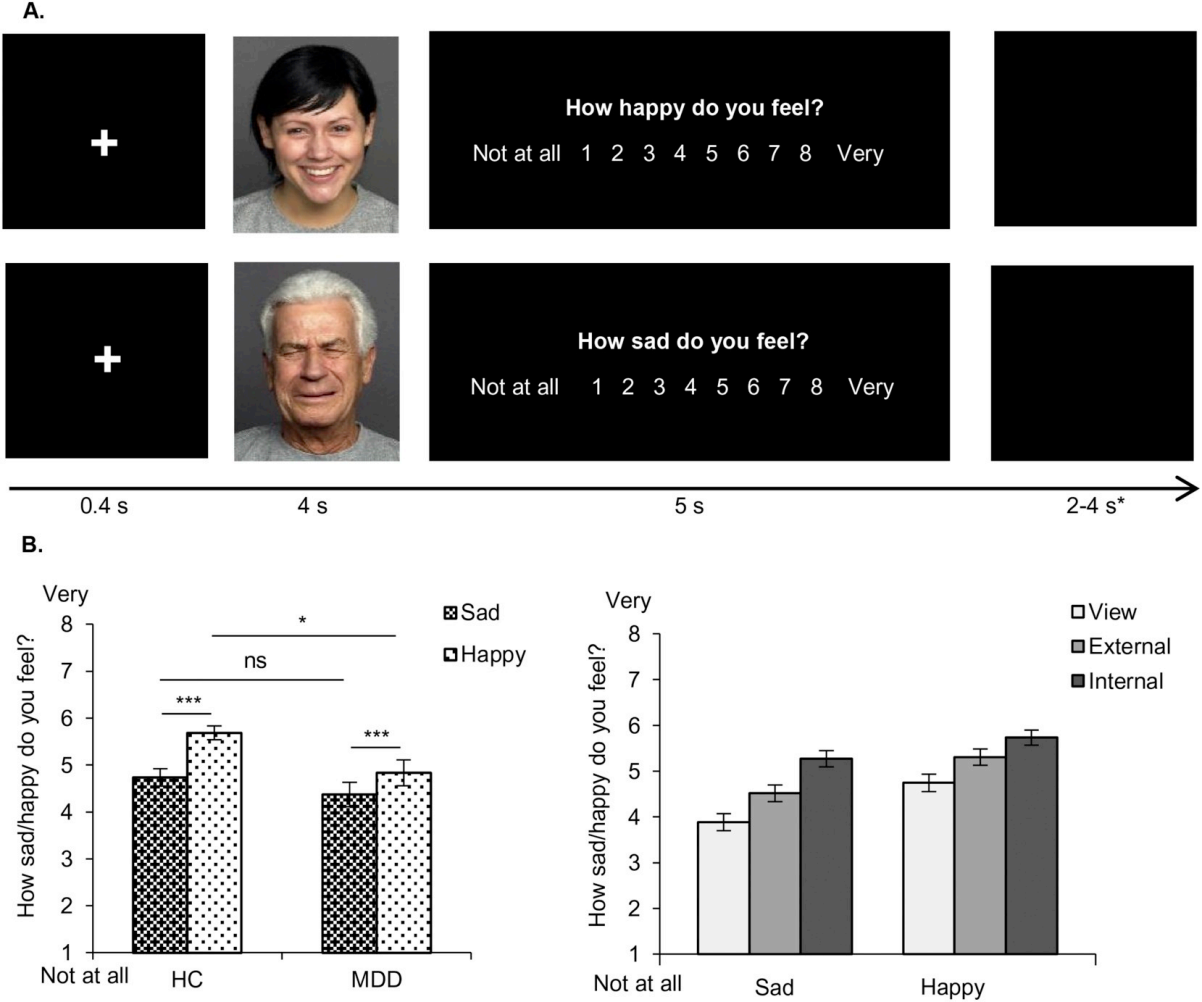
A. Emotion regulation task. Faces are presented for 4 s, followed by a rating phase of maximum 5 s. A 400 ms fixation cross primes the participant for the next face. Participants should indicate on a scale ranging from 1 ‘not at all’ to 8 ‘very’ how sad (regarding sad faces) and happy (regarding) happy faces they feel. They are instructed to apply no emotion regulation strategy in the control condition *view.* In two emotion regulation conditions *internal* and *external* they should imagine a person of close relation being depicted in the picture in order to increase personal relevance. In the *internal* condition, the participants are additionally instructed to imagine that the person in the picture is sad/happy because of them (internal attribution) whereas in the *external* condition they should imagine that they have nothing to do with the emotional state of the person (external attribution). Instructions are verbally and visually given at the beginning of each condition. *The inter-stimulus interval amounts to 2–4 s and is extended to 9–11 s after each mini-block, containing 5 faces of the same emotions. B. Mean ratings of the emotion regulation task (with standard errors). Left graph: group by emotion interaction (significant effects indicated with asterisks). Right graph: emotion by condition interaction (significant differences are not graphically indicated for visual purposes). * *p* ≤ .05, ** *p* ≤ .01, *** *p* ≤ .001.

Dysfunctional social behavior in depression (Hirschfeld et al., 1999) calls for investigating and applying emotion regulation in interpersonal interactions (Radke et al., 2018). Facial emotions are an important component of social communication, and therefore have a high ecologic validity and offer an ideal possibility to investigate emotion regulation in social situations. Therefore, pictures of 60 sad and 60 happy Caucasian faces from the FACES database (Ebner et al., 2010) were each presented for 4 s. During a rating phase of maximum 5 s participants indicated by a button press how sad (regarding sad faces) or happy (regarding happy faces) they felt on a scale ranging from 1 (“not at all”) to 8 (“very”). A 400 ms fixation cross primed the participants for the next face (Fig. 2A). Faces of the same emotion were grouped into mini-blocks, each consisting of 5 trials. The inter-stimulus interval within mini-blocks amounted to 2–4 s and between mini-blocks to 9–11 s.

The paradigm consisted of three counterbalanced conditions, implemented in three separate blocks. Each condition contained 20 sad (=4 mini-blocks) and 20 happy (=4 mini-blocks) faces. In the *view* condition, no regulation was applied. In the experimental conditions *internal* and *external*, participants applied causal attributions in order to regulate their emotions. In both conditions, participants were asked to imagine a person of close relation being depicted in the picture in order to increase personal relevance. In the *internal* condition, the participants were additionally instructed to imagine that the person in the picture was sad/happy because of them (internal attribution) whereas in the *external* condition they should imagine that they had nothing to do with the emotional state of the person (external attribution). Instructions were verbally and visually given at the beginning of each condition. Prior to the experiment, all participants performed several practice trials to ensure full comprehension of the task. Additionally, a qualitative assessment of the applied strategy was collected at the end of the experiment. This assessment included questions regarding the content of the imagined situations to ensure that participants correctly applied causal attributions. Furthermore, participants were asked how difficult the task was and how much effort they put into the task. A similar design has been successfully implemented in a previous study (Radke et al., 2018).

Stimuli were generated by Presentation Software (Neurobehavioral Systems, Albany, CA) and viewed on a screen at the end of the MR scanner through a mirror mounted on the head coil.

### 2.5 Image acquisition and preprocessing

MRI data was acquired on a 3 T Siemens PRISMA scanner at the Department of Psychiatry, Psychotherapy and Psychosomatics of the University Hospital RWTH Aachen using a 24-channel head coil. Functional data of the emotion regulation paradigm was obtained with a T2*-weighted echo-planar sequence (TR: 2000 ms, TE: 30 ms, FoV: 210 mm, 36 slices with ACPC orientation, voxel size: 3.3 × 3.3 × 3.0 mm, flip angle: 77°, distance factor: 20% (=0.6 mm)). Before the experimental paradigm, a resting-state scan with the same parameters and 210 volumes (7 min) was acquired. During this scan a fixation cross was presented and participants were instructed to keep the eyes open, not to fall asleep and to let their mind wander. Furthermore, an anatomical reference image was acquired with a sagittal T_1_-weighted 3D magnetization-prepared rapid gradient-echo (MPRAGE) sequence at the end of the scanning session (TR: 2300 ms, TE: 2.98 ms, FoV: 256 × 256 mm, voxel size: 1 × 1 × 1 mm, flip angle: 9%, distance factor: 50%).

For preprocessing of task-based functional data, statistical parametric mapping 12 (SPM12, Wellcome Department of Imaging Neuroscience, London) was used. In order to allow for magnetic field saturation, the first volumes of each condition were discarded. The remaining images were realigned to the mean image and slice-time corrected. Subsequently, a 3-step co-registration was conducted: (1) coregistration of the 3D-MPRAGE image to the 152 subject T1-weigthed template of SPM12, (2) coregistration of the (mean) EPI images to the EPI template, and (3) coregistration of the 3D-MPRAGE image to the EPI mean image. All images were normalized to the Montreal Neurological Institute (MNI) space, resampled to a resolution of 2 × 2 × 2 mm, and spatially smoothed using a 6 mm full-width-at-half-maximum Gaussian kernel.

Preprocessing of resting-state data was implemented in SPM12 as well as in the XCP Engine (Ciric et al., 2017), a multi-modal toolkit for the processing of neuro-images that employs processing instruments from frequently used software libraries, including FSL, ANTs, and AFNI. Data from 1 HC was discarded due to technical problems during data acquisition, resulting in 25 HC and 26 MDD for resting-state analysis. First, the initial 4 volumes were discarded to allow for equilibration of the magnetic field. Estimates of motion were then obtained using MCFLIRT (Jenkinson et al., 2002) to align functional images to a selected reference. Brain voxels were identified and extracted using BET (Jenkinson et al., 2005). Data were subsequently demeaned, and linear and quadratic trends were removed using a linear fit. Additionally, AFNI's 3dDespike utility was used to identify intensity outliers and to interpolate over these outliers. Subsequently, a deterministic 3D-MPRAGE image, consisting of white matter, grey matter, and cerebrospinal fluid, was created for each subject and co-registered to the mean functional image using SPM12. Preprocessed functional images, the mean functional image, and the co-registered deterministic 3D-MPRAGE image were normalized to MNI space using unified segmentation (Ashburner and Friston, 2005; SPM12). In order to account for subject movement, initial preprocessing was followed by confound regression using the XCP Engine and included nuisance parameters derived from six movement estimates and three physiological time series (mean time series in white matter and cerebrospinal fluid, mean global signal) as well as their temporal derivatives and quadratic expansions of the derivatives (36 parameters; Satterthwaite et al., 2013). Temporal filtering was performed using a bandpass filter of 0.01–0.08 Hz (first-order Butterworth filter; Hallquist et al., 2013) and images were smoothed in SUSAN using a Gaussian-weighted kernel with 6 mm FWHM (Smith and Brady, 1997).

### 2.6 Statistical analysis

#### 2.6.1 Clinical assessment

Clinical data was compared between groups using independent *t*-tests or Mann-Whitney-*U* tests if the data was not normally distributed (see Table 1 for the results).

#### 2.6.2 Trait attributional style (ASF-E)

In order to examine group differences in trait attributional style, mean scores of the ASF-E were analyzed with a repeated-measures ANOVA with group (HC, MDD) as between-subjects factor and valence (positive event, negative event) as within-subjects factor. Significant effects were followed-up with Bonferroni-corrected pairwise comparisons. To describe the relationship between trait attributional style (ASF-E) and depression severity (BDI-II, HRSD, MASQ), spearman correlations were computed.

#### 2.6.3 Instructed causal attributions (emotion regulation task) – Behavioral (emotion ratings)

Next, we investigated the influence of instructed causal attributions on subjective emotions. For this purpose, emotion ratings of the emotion regulation task were averaged and analyzed with a repeated-measures ANOVA with group (HC, MDD) as between-subjects factor and condition (view, external, internal) and emotion (sad, happy) as within-subjects factors. Due to violations of the assumptions of normal distribution and equal variances, the ratings were reverse coded and transformed using a logarithmic transformation (y = log10[x + 1]). Significant effects were followed-up with Bonferroni-corrected pairwise comparisons. To examine the relationship between subjective emotion ratings (view sad/happy, internal sad/happy, external sad/happy) and both depressive symptoms (BDI-II, HRSD, MASQ) and trait attributional style (ASF-E), spearman correlations were calculated.

Statistical testing was performed in SPSS 22.0 applying an α-level of *p* ≤ .05.

#### 2.6.4 Instructed causal attributions (emotion regulation task) – Neural (fMRI)

FMRI data was analyzed in SPM12. Using an event-related GLM model, events of interest were isolated by convolving vectors of stimulus onset times and stimulus duration (4 s) with the canonical hemodynamic response function. A first-level event-related GLM model was estimated with 6 regressors of interest (3 conditions by 2 emotions) and 7 regressors of no interest (rating phase, 6 movement parameters). Images were high-pass filtered at 128 s and an autoregressive AR(1) model was used to account for temporal autocorrelations.

On a group level, a full-factorial GLM-analysis with the factors group, condition, and emotion was conducted. In order to examine emotion regulation ability, emotion regulation conditions are typically compared to no-regulation conditions (Kanske et al., 2012; Morawetz et al., 2017). For this reason, we compared the internal and external condition to the view condition in order to investigate neural correlates of emotion regulation ability. Furthermore, a particular strength of this study is the assessment of the regulation of both positive and negative emotions. We therefore analyzed emotion-specific regulation effects (i.e. upregulation of positive emotions: internal happy > external happy; downregulation of negative emotions: external sad > internal sad).

In order to examine whether neural attribution effects (mean beta estimates across voxels of identified clusters) are associated with depression severity (BDI-II, HRSD, MASQ) and trait attributional style (ASF-E), spearman correlation were calculated in SPSS 22.0.

We corrected for whole-brain multiple comparisons using a Monte-Carlo correction (Forman et al., 1995; voxel-level threshold: *p* < .001, cluster-level threshold: *p* < .05, smoothness: 9.0 × 9.0 × 8.8 mm, 10,000 iterations) implemented in AFNI's 3dClustSim.

#### 2.6.5 Connectivity (seed-based resting-state fMRI)

We applied a seed-region approach (Biswal et al., 1995) using the XCP Engine (Ciric et al., 2017) to examine whole-brain functional connectivity during resting-state and defined a seed region based on fMRI results (i.e. activated brain region during the emotion regulation task). A 5 mm spherical seed at the left precuneus (MNI: −6 -55 33 [averaged coordinates of external > view and internal > view]) was created, since the left precuneus was activated during both regulation conditions. By examining whole-brain connectivity of the precuneus, we could detect whether connectivity between only emotion regulation regions is affected in MDD or whether connectivity with other regions is impaired as well. Linear correlation coefficients between time series of the seed region and all other voxels of the brain were calculated and transformed into Fisher's *Z*-scores. Groups were compared in a full-factorial GLM-analysis using SPM12. Furthermore, we examined the relationship between whole-brain precuneus connectivity and trait attributional style (ASF-E) using a whole-brain simple regression in SPM12. As above, multiple comparisons were corrected applying a Monte-Carlo correction (Forman et al., 1995; voxel-level threshold: *p* < .001, cluster-level threshold: *p* < .05, smoothness: 6.1 × 6.2 × 6.1 mm, 10,000 iterations) implemented in AFNI's 3dClustSim.

## 3 Results

### 3.1 Trait attributional style (ASF-E)

**Fig. 3:**
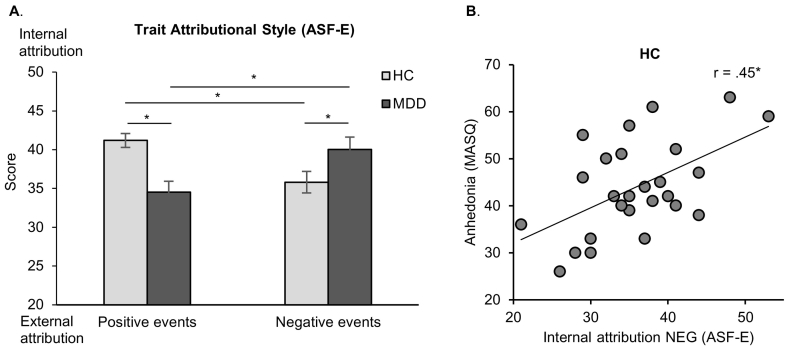
A. Trait attributional style (ASF-E; mean scores with standard errors) in patients with major depressive disorder (MDD) and healthy controls (HC). MDD show less internal attributions of positive events and more internal attributions of negative events than HC. Furthermore, MDD show more internal attributions of negative compared to positive events. By contrast, HC show more internal attributions of positive compared to negative events. B. Spearman correlation between trait attributional style (ASF-E: internal attributions of negative events) and anhedonia (MASQ) in HC. * *p* ≤ .05, ** *p* ≤ .01, *** *p* ≤ .001.

Repeated-measures ANOVA revealed a non-significant main effect of group (*F*(1,50) = 1.105, *p* = .298, *ƞ*_*p*_^*2*^ = 0.022) and a non-significant main effect of valence (*F*(1,50) = 0.001, *p* = .970, *ƞ*_*p*_^*2*^ < 0.001). A significant group by valence interaction (*F*(1,50) = 13.12, *p* = .001, *ƞ*_*p*_^*2*^ = 0.21) showed that MDD indeed have a non-self-serving trait attributional style. Bonferroni-corrected pairwise comparisons confirmed more internal attributions of negative events (*p* = .050) and less internal attributions of positive events (*p* < .001) in MDD compared to HC. Furthermore, patients showed more internal attributions of negative events compared to positive events (*p* = .013), while controls showed more internal attributions of positive events compared to negative events (*p* = .014; Fig. 3A). Due to significant group differences in trait attributional style (ASF-E) and clinical parameters (BDI-II, MASQ), correlation analyses were separately performed for MDD and HC. Results revealed that HC with increasing anhedonia (MASQ) showed more internal attributions of negative events (*r* = 0.45, *p* = .020; Fig. 3B). No further correlation between trait attributional style and depression severity reached significance (all *p* ≥ .083).

### 3.2 Instructed causal attributions (emotion regulation task)

#### 3.2.1 Behavioral (subjective emotion ratings)

A repeated-measures ANOVA revealed differences in subjective emotion ratings between conditions (main effect condition: *F*(2,100) = 53.63, *p* < .001, *ƞ*_*p*_^*2*^ = 0.52; see Table 2 for mean ratings), confirming that instructed causal attributions successfully regulated emotions. Bonferroni-corrected pairwise comparisons showed that emotion ratings in all three conditions differed significantly from each other (all *p* < .001). Furthermore, participants generally reported higher happiness than sadness ratings (main effect emotion: *F*(1,50) = 57.10, *p* < .001, *ƞ*_*p*_^*2*^ = 0.53). Groups did not differ significantly in their overall emotion ratings (main effect group: *F*(1,50) = 3.42, *p* = .070, *ƞ*_*p*_^*2*^ = 0.064).

**Table 2:** Mean emotion ratings of the emotion regulation task.

Condition	Group	Mean rating	SD
View Total	HC	4.64	1.02
	MDD	3.99	1.40
	Total	4.31	1.25
View Sad	HC	4.07	1.22
	MDD	3.69	1.46
	Total	3.88	1.35
View Happy	HC	5.21	1.04
	MDD	4.28	1.52
	Total	4.75	1.37
Internal Total	HC	5.80	0.94
	MDD	5.20	1.30
	Total	5.50	1.17
Internal Sad	HC	5.50	1.18
	MDD	5.03	1.34
	Total	5.27	1.27
Internal Happy	HC	6.10	0.75
	MDD	5.37	1.43
	Total	5.73	1.19
External Total	HC	5.19	0.87
	MDD	4.63	1.43
	Total	4.91	1.21
External Sad	HC	4.64	1.06
	MDD	4.39	1.49
	Total	4.51	1.29
External Happy	HC	5.75	0.84
	MDD	4.86	1.46
	Total	5.31	1.26
Happy	HC	5.69	0.77
	MDD	4.84	1.40
	Total	5.26	1.20
Sad	HC	4.74	0.94
	MDD	4.37	1.34
	Total	4.55	1.16

Mean emotion ratings with standard deviations (SD). Abbreviations: HC = healthy controls, MDD = patients with major depressive disorder.

Following-up the significant emotion x group interaction (*F*(1,50) = 6.98, *p* = .011, *ƞ*_*p*_^*2*^ = 0.12; Fig. 2B, left graph) with pairwise comparisons revealed higher happiness ratings in HC compared to MDD (*p* = .017), whereas sadness ratings did not differ between groups (*p* = .315). Happiness ratings were significantly higher than sadness ratings in both groups (HC: *p* < .001; MDD: *p* = .001). Follow-up pairwise comparisons of the significant condition x emotion interaction (*F*(1,50) = 3.15, *p* = .047, *ƞ*_*p*_^*2*^ = 0.059; Fig. 2B, right graph) revealed that all three conditions differed from each other when compared separately for sadness and happiness (all *p* ≤ .001). Similarly, sadness and happiness ratings differed from each other when compared separately for each condition (all *p* < .001). Additionally conducted within-subject contrasts revealed that sadness ratings were more strongly down-regulated than happiness ratings during external compared to internal attribution (*p* = .023). There was no further significant interaction (group x condition interaction: *F*(2,100) = 0.182, *p* = .834 *ƞ*_*p*_^*2*^ < 0.01; group x condition x emotion interaction: *F*(2,100) = 2.44, *p* = .092, *ƞ*_*p*_^*2*^ = 0.05).

After examining the influence of instructed causal attributions on subjective emotions, we explored the relationship between subjective emotions and both trait attributional style (ASF-E) and depression severity (BDI-II, MASQ, HDSR) using spearman correlations. Due to significant group differences in trait attributional style and clinical parameters, spearman correlations were calculated per group. Within HC, happiness ratings during *internal positive* (emotion regulation task) correlated negatively with the tendency to relate causes of negative events to the self (ASF-E; *r* = −0.429, *p* = .029) as well as with anhedonia (MASQ; *r* = −0.440, *p* = .024). Thus, HC with a non-adaptive attributional style and anhedonia symptomes reported lower happiness ratings. All other correlations in HC were not significant (all *p* ≥ .083).

Within MDD, no significant correlation emerged between subjective emotion ratings and neither trait attributional style, nor depressive symptoms (all *p* ≥ .316).

#### 3.2.2 Neural (fMRI; Table 3)

##### 3.2.2.1 Across groups (whole brain)

**Fig. 4:**
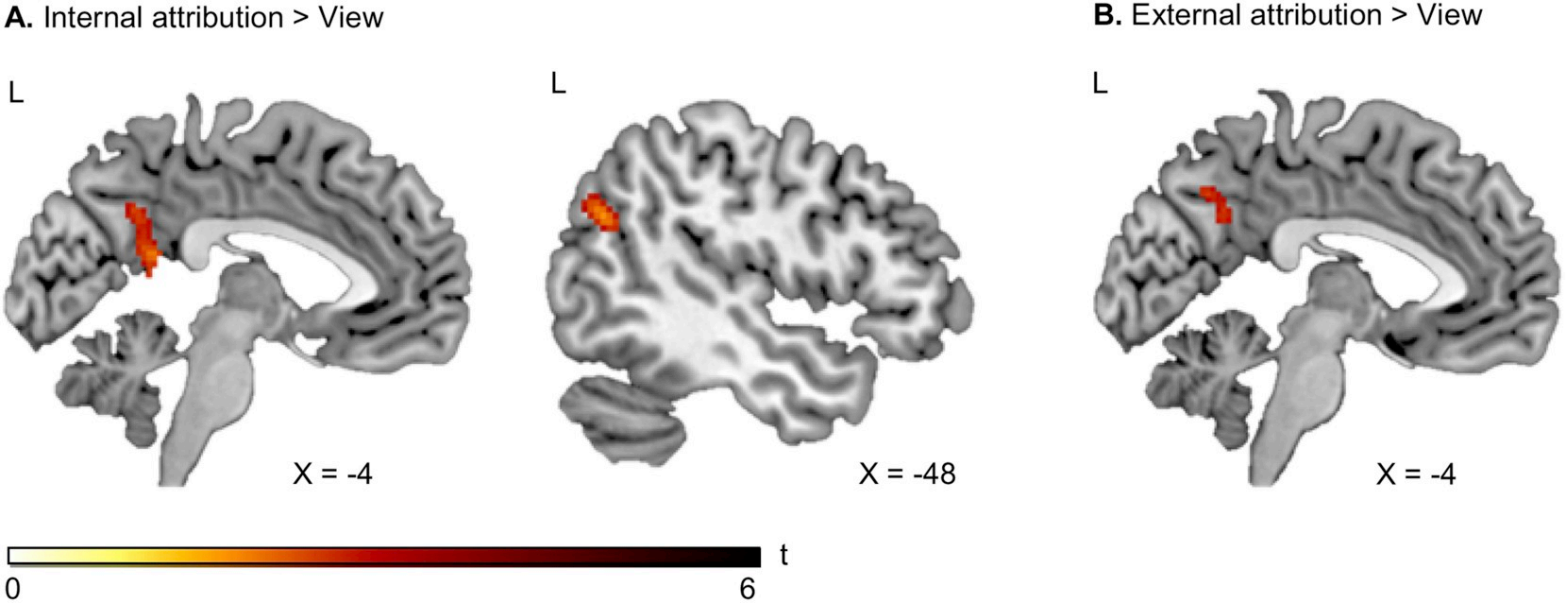
A. Across groups, internal attributions compared to view engage left precuneus and left angular gyrus (Monte-Carlo cluster-level corrected). B. Across groups, external attributions compared to view are associated with activation in the left precuneus (Monte-Carlo cluster-level corrected).

Besides effects of instructed causal attribution on subjective emotions, we examined its neural correlates using a full-factorial GLM. Internal attributions compared to view engaged the left precuneus and left angular gyrus (Fig. 4A). Similarly, external attributions compared to view revealed significant activation in the left precuneus (Fig. 4B). The opposite contrasts (view>internal, view>external) yielded no suprathreshold activation. Likewise, emotion-specific contrasts (i.e. internal compared to external attributions of positive events, external attribution compared to internal attribution of negative events) yielded no suprathreshold activation.

**Table 3:** Whole brain effects of the emotion regulation task.

	k	Side	MNI			t-value
			x	y	z	
HC + MDD						
Internal>View						
Precuneus	153	L	−6	−54	34	4.06
2. Maximum: PCC		L	−4	−48	18	4.04
Angular Gyrus	124	L	−48	−64	28	4.27
View>Internal		*No suprathreshold activation*				
External>View						
Precuneus	100	L	−6	−56	32	3.77
View>External		*No suprathreshold activation*				
Internal Happy>External Happy		*No suprathreshold activation*				
External Sad>Internal Sad		*No suprathreshold activation*				

HC > MDD						
View		*No suprathreshold activation*				
Internal						
Lingual Gyrus	266	R	8	−76	2	4.77
Lingual Gyrus	118	L	−8	−76	−6	4.47
External		*No suprathreshold activation*				
Internal vs. View		*No suprathreshold activation*				
External vs. View		*No suprathreshold activation*				
Internal Positive>External Positive						
Lingual Gyrus	152	L	−12	−52	−4	4.19
External Negative>Internal Negative		*No suprathreshold activation*				

MDD > HC						
View						
Fusiform Gyrus	106	L	−18	−88	−8	5.95
Internal						
Fusiform Gyrus	99	L	−18	−88	−8	5.26
External						
Lingual Gyrus	145	L	−18	−86	−8	5.89
Fusiform Gyrus	120	L	−40	−64	−18	4.17
Fusiform Gyrus	100	R	30	−80	−10	5.71
Fusiform Gyrus	95	R	32	−48	−18	4.79
Internal vs. View		*No suprathreshold activation*				
External vs. View		*No suprathreshold activation*				
Internal Positive>External Positive		*No suprathreshold activation*				
External Negative>Internal Negative		*No suprathreshold activation*				

HC						
Internal>View						
Middle Temporal Gyrus	135	L	−52	−14	−12	
Lingual Gyrus	91	L	−10	−54	0	3.88
View>Internal		*No suprathreshold activation*				
External vs. View		*No suprathreshold activation*				
Internal Positive>External Positive						
Parahippocampal Gyrus	190	L	−18	−40	−4	4.58
External Negative>Internal Negative		*No suprathreshold activation*				

MDD						
Internal vs. View		*No suprathreshold activation*				
External>View						
Precuneus/PCC	92	L	−4	−60	18	4.33
View>External		*No suprathreshold activation*				
Internal Positive>External Positive		*No suprathreshold activation*				
External Negative>Internal Negative						
Superior Frontal Gyrus	209	R	20	22	42	4.77

Whole-brain effects across groups, between groups, and within groups for the emotion regulation task. Results are Monte-Carlo corrected and cluster size (k), side, MNI coordinates and t-values are given.

*Note*: HC = Healthy controls; MDD = Patients with major depressive disorder; L = Left; R = Right; PCC = Posterior Cingulate Cortex.

##### 3.2.2.2 Between groups (whole brain)

Next, we tested whether there would be group differences in brain regions linked to instructed causal attributions. HC compared to MDD showed increased activation in the right lingual gyrus during internal attributions and increased activation in the left lingual gyrus during internal positive > external positive. MDD compared to HC showed a stronger recruitment of the left fusiform gyrus in the view, internal and external attribution condition. Additionally, MDD engaged the right fusiform gyrus more strongly than HC during external attributions. No further group comparisons yielded suprathreshold activation.

##### 3.2.2.3 Within groups (whole brain)

Based on the previously identified group differences, we further explored group-specific effects of causal attributions. In HC, internal attributions compared to view revealed activation in the left middle temporal gyrus and left lingual gyrus, whereas external attributions (compared to view) showed no suprathreshold activations. The control condition view yielded no significant activation when compared to internal or external attribution. Regarding emotion-specific effects, we found left parahippocampal gyrus activation in HC during internal compared to external attributions of positive events, whereas external compared to internal attributions of negative events yielded no suprathreshold activation.

**Fig. 5:**
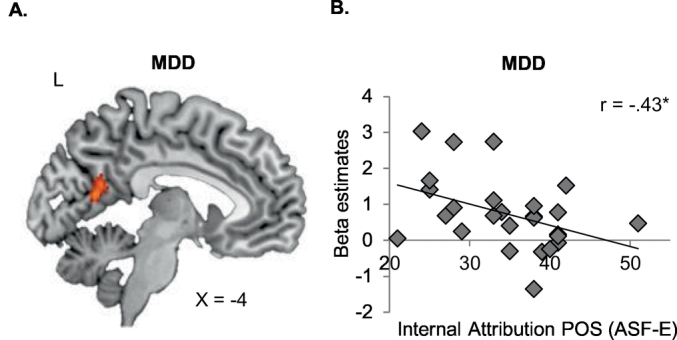
A. Within MDD, external attributions compared to view are associated with activation in the left precuneus/left posterior cingulate cortex (PCC; Monte-Carlo cluster-level corrected). B. *Beta* estimates of the left precuneus/PCC cluster for external>view correlate negatively with internal attributions of positive events (ASF-E). * *p* ≤ .05, ** *p* ≤ .01, *** *p* ≤ .001.

In MDD, comparing internal attribution to view resulted in no suprathreshold activation, while external attribution (compared to view) showed stronger engagement of the left precuneus, extending to the left posterior cingulate cortex (PCC; Fig. 5). Similar to HC, in MDD the view condition yielded no suprathreshold activation when compared to internal or external attribution. With respect to emotion-specific contrasts we identified increased activation in MDD in the right superior frontal gyrus during external compared to internal attributions of negative events, while internal compared to external attributions of positive events yielded no suprathreshold activation.

##### 3.2.2.4 Correlations with trait attributional style and depressive symptoms

**Fig. 6:**
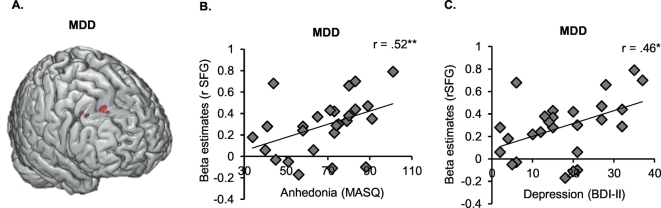
A. Within patients with major depressive disorder (MDD), external compared to internal attributions of negative events activates the right superior frontal gyrus (rSFG). B. Activation in the rSFG correlates positively with anhedonia (MASQ). C. Activation in the rSFG correlates positively with depression symptoms (BDI-II).

Having established the whole brain neural correlates of instructed causal attributions we examined whether brain activation in the identified clusters would be associated with trait attributional style (ASF-E) and depression severity (BDI-II, HRSD, MASQ). Due to significant group differences in attributional style and clinical parameters, spearman correlations were separately calculated per group. Otherwise, significant correlations might merely be driven by significant group differences in attributional style and depression symptoms. Within HC, no significant correlations emerged (all *p* ≥ .151). Within MDD, more activation in the left precuneus during external attribution (compared to view) was linked to less internal attributions of positive events (ASF-E; *r* = −0.43, *p* = .027; Fig. 5B). Furthermore, within MDD, activation in the right superior frontal gyrus during external compared to internal attributions of negative events correlated significantly with depression severity (BDI-II: *r* = 0.458, *p* = .019; MASQ: *r* = 0.515, *p* = .007; Fig. 6). All other correlations were non-significant (all *p* ≥ .117).

### 3.3 Connectivity (seed-based resting-state fMRI)

#### 3.3.1 Between groups

To extend our findings of attribution-related precuneus activity, we explored group differences in whole-brain connectivity of this particular region. Across groups, the left precuneus seed was functionally connected to the default mode network, but no suprathreshold differences emerged between groups.

#### 3.3.2 Relationship connectivity and trait attributional style

**Fig. 7:**
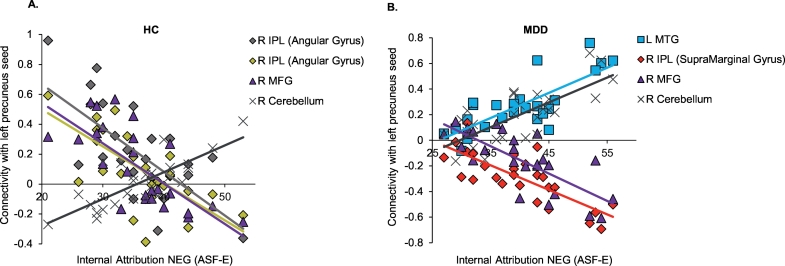
A. Within HC, there was a negative association between internal attributions of negative events (ASF-E) and connectivity of the left precuneus with two clusters in the right inferior parietal lobule (IPL) and with the right middle frontal gyrus (MFG). A positive association emerged between internal attributions of negative events (ASF-E) and left precuneus – right cerebellum connectivity. B. Within MDD, there was a positive association between internal attributions of negative events (ASF-E) and the left precuneus – left middle temporal gyrus (MTG) and left precuneus – right cerebellum connectivity. A negative association emerged between internal attributions of negative events (ASF-E) and left precuneus – right supramarginal gyrus connectivity as well as left precuneus – right middle frontal gyrus (MFG) connectivity.

Finally, we were interested whether connectivity of the precuneus, which is particularly engaged during instructed attributions, was related to trait attributional style (ASF-E). Due to significant group differences in attributional style (ASF-E), simple regressions were calculated separately per group (Table 4). Within HC, more internal attributions of positive events (ASF-E) were associated with decreased connectivity within the left precuneus. Moreover, internal attributions of negative events (ASF-E) were positively associated with left precuneus – right cerebellum connectivity and negatively associated with left precuneus – right inferior parietal lobule (IPL; angular gyrus) connectivity as well as left precuneus – right middle frontal gyrus (MFG) connectivity (Fig. 7A).

**Table 4:** Relationship between resting-state-fMRI connectivity and trait attributional style (ASF-E).

Connectivity	Association with trait attributional style (ASF-E)	k	MNI			t-value
HC			x	y	z	
L Precuneus - R Cerebellum	+ NEG Internal	165	50	−58	−52	5.63
L Precuneus - R IPL (AG)	- NEG Internal	119	54	−52	42	5.07
L Precuneus - R MFG	- NEG Internal	94	36	14	56	5.19
L Precuneus - R IPL (AG)	- NEG Internal	90	40	−54	42	5.11
L Precuneus - L Precuneus	- POS Internal	60	−2	−50	42	5.44
						
MDD						
L Precuneus - R Cerebellum	+ NEG Internal	296	14	−88	−34	6.20
L Precuneus - R IPL (SMG)	- NEG Internal	77	58	−28	34	4.86
L Precuneus - L MTG	+ NEG Internal	68	−56	−2	−24	5.18
L Precuneus - R MFG	- NEG Internal	65	30	50	22	5.72

Significant association between left precuneus [−6–55 33] connectivity and trait attributional style (ASF-E) in resting-state. Results are Monte-Carlo corrected and cluster size (k), side, MNI coordinates and t-values are given.

Abbreviations: AG = angular gyrus; ASF-E = attributional style questionnaire for adults; HC = Healthy controls; IPL = inferior parietal lobule; L = left; MDD = Patients with major depressive disorder; MFG = middle frontal gyrus; MTG = middle temporal gyrus; NEG Internal = internal attributions of negative events; POS Internal = internal attributions of positive events; R = right; SMA = supramarginal gyrus; + = positive association; − = negative association.

Within MDD, internal attributions of negative events (ASF-E) were positively associated with left precuneus – left cerebellum and left precuneus – left middle temporal gyrus (MTG) connectivity. A negative association emerged between internal attributions of negative events (ASF-E) and left precuneus – right IPL (supramarginal gyrus) and left precuneus – right MFG connectivity (Fig. 7B).

## 4 Discussion

According to the World Health Organization, depression is currently the leading cause of disability worldwide (World Health Organization, 2017). Since depression is characterized by disrupted neural and behavioral emotion regulation as well as a dysfunctional trait attributional style, the current fMRI study examined for the first time how instructed causal attributions of positive and negative events affect emotions in patients with lifetime major depressive disorder and healthy controls. Behaviorally, patients compared to controls displayed a non-self-serving trait attributional style, reflected in fewer internal attributions of positive and more internal attributions of negative events. However, when instructed to apply causal attributions, both patients and controls were able to successfully regulate sadness and happiness on a behavioral and neural level. Across groups, both internal and external causal attributions were linked to increased precuneus activation, which correlated with patients' trait attributional style. Furthermore, intentionally relating causes of positive events to the self (instead of to external sources) engaged the left parahippocampal gyrus in controls, while attributing causes of negative events to external sources (instead of to the self) recruited the right superior frontal gyrus in patients. Superior frontal activation in patients was positively related to depression severity. Interestingly, resting-state connectivity of the precuneus with emotion regulation brain regions was related to an adaptive trait attributional style in patients and controls.

### 4.1 Instructed causal attributions

Causal attributions recruited brain regions involved in self-related processing and emotion regulation. Across patients and controls, both external and internal attributions (compared to view) recruited the left precuneus, and internal attributions additionally engaged the left angular gyrus. The precuneus is involved in numerous processes, including reflective self-awareness (Kjaer et al., 2002), perspective taking (Ruby and Decety, 2001), social inferential processing (Kuzmanovic et al., 2012), observation of social interactions (Iacoboni et al., 2004), episodic memory (Lundstrom et al., 2005), and most importantly, in causal attributions (Cabanis et al., 2013; Fukushima et al., 2013; Grimm et al., 2009; Kestemont et al., 2015; Seidel et al., 2010). Therefore, precuneus activation in the current study might reflect the attempt to relate to a partner in a social interaction and to modulate self-responsibility for that situation. Separate subregions of the precuneus might be associated with different processes (Cabanis et al., 2013; Margulies et al., 2009), as an anterior, central, and posterior subdivision, were clearly differentiated during resting-state (Margulies et al., 2009). The central subdivision, to which the precuneus cluster identified in the current study belongs, is functionally connected to cognitive/associative brain regions (i.e. angular gyrus, dorsolateral prefrontal cortex). These functional connections facilitate interaction with cognitive control regions, as indicated by the involvement of the precuneus in cognitive emotion regulation (Morawetz et al., 2016). By contrast to instructed attributions, free attributions, as examined in Seidel et al. (2010), recruited a more ventral subdivision of the precuneus which is rather connected to limbic structures (e.g. anterior cingulate, paracingulate, and medial prefrotal cortex; Margulies et al., 2009). The engagement of the precuneus during free attributions might therefore reflect the experienced emotion instead of cognitive control. Other studies on free attributions, however, revealed precuneus cluster of the central subdivision (Cabanis et al., 2013; Kestemont et al., 2015), which emphasizes the need of further studies comparing free and instructed causal attributions.

Intriguingly, in the current study, patients with a more self-serving attributional style (i.e. relating positive events more to themselves) showed less precuneus/PCC activation during external attributions [external>view]. Thus, lower engagement of the precuneus/PCC might represent the success of intentionally relating causes of events to external sources. This necessary skill in daily life for regulating emotions may therefore be linked to a generally healthier cognitive style. Besides the precuneus, internal attributions recruited the angular gyrus across patients and controls. The angular gyrus is claimed to be an “executive arm” of cognitive emotion regulation (Kohn et al., 2014). Since the angular gyrus is associated with self-relevant internal cognitive processes, semantic processes, and episodic memory (Andrews-Hanna et al., 2010; Humphreys and Lambon Ralph, 2015), it is suggested to regulate emotions by generating imagined or remembered situations (Kohn et al., 2014).

Besides general effects of instructed attributions (i.e. attribution > view), we were further interested in emotion-specific attribution effects. Intentionally relating causes of positive events to the self (instead of to external sources) recruited the left parahippocampal gyrus in healthy controls. Due to its involvement in memory retrieval (Maguire and Mummery, 1999), such parahippocampal engagement might indicate successful retrieval of positive/happy memories, which in turn might up-regulate positive emotions. Relating causes of negative events to external sources (instead of to the self), in turn, recruited the superior frontal gyrus in depressed patients. The superior frontal gyrus is part of the cognitive emotion regulation network (Frank et al., 2014; Morawetz et al., 2017) and suggested to be involved in sustained cognitive aspects of emotion regulation (Frank et al., 2014). The positive association between superior frontal activation and depression severity might imply that the more depressed patients are, the more cognitive resources they need in order to down-regulate negative emotions.

Interestingly, in contrast to emotion regulation studies using emotional scenes as stimuli (e.g. IAPS pictures), we particularly observed brain activation in the precuneus during emotion regulation. Morawetz et al. (2016) argue that interpersonal emotion regulation might rely to a greater extent on cognitive functions such as self-reflection and mentalizing than the regulation of complex scenes and therefore engages different brain regions. Furthermore, participants had explicit instructions to apply causal attributions, which rely on the precuneus, whereas usual emotion regulation studies apply a variety of different tactics.

### 4.2 Connectivity

During resting-state, precuneus connectivity was associated with attributional style (ASF-E). In patients and in controls, stronger connectivity between the left precuneus and the well-established emotion regulation regions IPL and MFG (Frank et al., 2014; Kohn et al., 2014; Carmen Morawetz et al., 2017) was related to a more self-serving attributional style (i.e. relating causes of negative events to external sources). This is in line with previous findings revealing a link between impaired connectivity between cognitive control regions and a dysfunctional attributional style (Stange et al., 2017). Adequate communication between the precuneus and regulation regions, at least during resting-state, therefore might facilitate an adaptive attributional style.

### 4.3 Group differences

The absence of group differences in brain regions linked to cognitive emotion regulation (e.g. prefrontal cortex) or attribution (e.g. precuneus) suggests that both controls and patients were able to regulate negative and positive emotions by applying causal attributions, which was further supported by subjective emotion ratings. Intact subjective (Kanske et al., 2012; Sheline et al., 2009) or neural (Dillon and Pizzagalli, 2013) emotion regulation has been previously reported in acute/unmedicated and remitted/medicated MDD, and in acute/unmedicated MDD, respectively. The lack of group differences in resting-state connectivity of the precuneus further points to adequate neural communication in our patient sample. Despite patients' ability to regulate emotions by applying causal attributions when instructed, they showed a dysfunctional trait attributional style (i.e. relating negative events to themselves and positive events to external sources in daily life). Mood and psychopathology are linked to individuals' causal attribution tendencies, and such non-self-serving attributions create vulnerability to depression (Abramson et al., 1978). Along these lines, the current study revealed a positive association between non-self-serving attributions (i.e. internal attributions of negative events) and symptom severity, namely anhedonia, in HC. Furthermore, HC with a non-self-serving attributional style (i.e. relating causes of negative events to the self) reported lower happiness ratings during emotion regulation. This emphasizes the importance of an adaptive attributional style for preserving mental health.

Despite intact emotion regulation, altered recruitment of extrastriate brain areas in MDD compared to HC implies generally disturbed processing of (facial) emotions in MDD, which seems characteristic for depression (Cusi et al., 2012). The processing of positive emotions seems to be particularly impaired in depression, as indicated by altered extrastriate engagement in patients compared to controls during internal attributions of positive events. Furthermore, subjective emotion ratings point to impaired processing of positive emotions in depression: patients and controls were similarly responsive to sad faces, but patients provided generally lower happiness ratings. Such a reduced reactivity to positive emotions is mirrored by higher anhedonia symptoms and is in line with previous research (Bylsma et al., 2008). Emotional disengagement and deficient reward processing observed in MDD (Proudfit et al., 2015) necessitate assessing not only negative but also positive emotion regulation in depression. Since patients frequently still suffer from a reduction of positive affect after treatment (Taylor et al., 2010), patients' well-being might be increased by addressing dysfunctional causal attributions. For instance, the positive psychology intervention ‘three good things’, which includes writing a diary of positive events and their causes, has been shown to successfully decrease depression and increase happiness (Seligman et al., 2005).

### 4.4 Limitations

In order to represent a broad spectrum of depression, the current study included both acutely depressed and (partially) remitted depressed patients since dysfunctional emotion regulation as well as attenuated resting-state connectivity is not only characteristic for acute depression, but also prevalent in remitted depression (e.g. Kanske et al., 2012; Stange et al., 2017). Missing correlations between symptoms of acute depression and emotion regulation ability further indicate that the ability to regulate emotions is not solely restricted to acute illness. Nonetheless, combining these two groups might have created substantial variation within the patient sample, which may have obscured regulation effects. Future research should directly compare individuals with a remitted and acute major depressive episode.

Furthermore, the majority of our patients received antidepressant medication at the time of testing. Previous research comparing depressed patients with different medication and illness statuses showed inconsistent results regarding emotion regulation ability. Smoski et al. (2013) for instance revealed disturbed neural activation in non-medicated, remitted patients. Neural alterations during emotion regulation has been also shown in medicated, acutely depressed patients (Erk et al., 2010). On the other hand, Dillon and Pizzagalli (2013) could not identify differences in emotion regulation between unmedicated, acute depressed patients and healthy controls. The role of medication in emotion regulation processes is thus still to be fully understood. But previous research suggests at least that neural emotion regulation effects in depression are not only due to patients' medication status. With respect to resting-state, antidepressant medication seems to alter functional connectivity. McCabe and Mishor (2011), for instance, revealed reduced subcortical-cortical resting-state functional connectivity in healthy participants after a 7-day SSRI and SNRI intake. Similarly, functional connectivity strength (FCS) changed after an 8-week intake of escitalopram (SSRI) in MDD (i.e. FCS increased in dorsomedial prefrontal cortex, FCS decreased in hippocampus; Wang et al., 2015). For this reason, it is important for future studies to examine resting-state connectivity of emotion regulation brain regions in non-medicated patients.

### 4.5 Conclusion and future directions

Despite these limitations, the current study provides new insights into the neural correlates of emotion regulation in major depression. Patients compared to controls showed a non-self-serving attributional style. However when instructed to apply causal attributions in an emotion regulation task, they were able to regulate negative and positive emotions via engagement of the precuneus. These findings emphasize that applying causal attributions might be a powerful emotion regulation strategy in depression. The results of the current study emphasize the need to study the regulation of both positive and negative emotions. In particular, the regulation of positive emotions has hardly been researched, but reveals important insights into the psychopathology and possible treatment approaches for depression. The direction of regulation (upregulation, downregulation) thereby depends on the valence of the emotion. In addition, the current study provides researchers with a new reappraisal strategy (i.e. instructed causal attributions) thereby complementing traditional strategies with a tactic which can be applied very well in daily life and in various (social) situations.

Future studies should investigate whether causal attributions reduce depressive symptoms (e.g. negative affect, anhedonia) and how emotion regulation by causal attributions can be applied in clinical practice. For example, educating patients about the influence of causal attributions on emotions could be incorporated in psychotherapy. Furthermore, by means of attribution trainings (e.g. via smartphones, group-therapy sessions) adaptive causal attributions could then be trained. Attributional retraining programs have already been shown to positively influence attributions in college students (Hall et al., 2011) and might be extended to therapy settings.
